# Supplementary material for: Disrupted Gray Matter Networks Associated with Cognitive Dysfunction in Cerebral Small Vessel Disease
Source: Brain Sci. 2023 Sep 22;13(10):1359. doi: 10.3390/brainsci13101359 (PMC10605932; doi:10.3390/brainsci13101359)
Supplement: Supplementary file 1 [file brainsci-13-01359-s001.zip › brainsci-2588272-supplementary.pdf]

Supplementary Table S1. The 90 cortical and subcortical regions of interest defined in our study.

**Regions**	**Abbr.**	**Regions**	**Abbr.**
Precentral gyrus	PreCG	Lingual gyrus	LING
Superior frontal gyrus, dorsolateral	SFGdor	Superior occipital gyrus	SOG
Superior frontal gyrus, orbital part	ORBsup	Middle occipital gyrus	MOG
Middle frontal gyrus	MFG	Inferior occipital gyrus	IOG
Middle frontal gyrus orbital part	ORBmid	Fusiform gyrus	FFG
Inferior frontal gyrus, opercular part	IFGoperc	Postcentral gyrus	PoCG
Inferior frontal gyrus, triangular part	IFGtriang	Superior parietal gyrus	SPG
Inferior frontal gyrus, orbital part	ORBinf	Inferior parietal, but supramarginal and angular gyri	IPL
Rolandic operculum	ROL	Supramarginal gyrus	SMG
Supplementary motor area	SMA	Angular gyrus	ANG
Olfactory cortex	OLF	Precuneus	PCUN
Superior frontal gyrus, medial	SFGmed	Paracentral lobule	PCL
Superior frontal gyrus, medial orbital	ORBsupmed	Caudate nucleus	CAU
Gyrus rectus	REC	Lenticular nucleus, putamen	PUT
Insula	INS	Lenticular nucleus, pallidum	PAL
Anterior cingulate and paracingulate gyri	ACG	Thalamus	THA
Median cingulate and paracingulate gyri	DCG	Heschl gyrus	HES
Posterior cingulate gyrus	PCG	Superior temporal gyrus	STG
Hippocampus	HIP	Temporal pole: superior temporal gyrus	TPOsup
Parahippocampal gyrus	PHG	Middle temporal gyrus	MTG
Amygdala	AMYG	Temporal pole: middle temporal gyrus	TPOmid
Calcarine fissure and surrounding cortex	CAL	Inferior temporal gyrus	ITG
Cuneus	CUN		

Supplementary Table S2. Global and local topological properties used in the study.

**Global network properties**	
**Global Efficiency E_{glob}**	E_{glob} is defined as the mean value of all regions' global efficiency.
**Local Efficiency E_{loc}**	E_{loc} is defined as the mean value of all regions' local efficiency.
**Shortest path length L_p**	L_p is defined as the average length of the shortest path between every two nodes in network G, which quantifies the ability for information to be propagated in parallel.
**Clustering coefficient C_p**	C_p is the average clustering coefficient over all nodes, which indicates the extent of local interconnectivity or cliquishness in a network
**Normalized L_p (λ)**	$\lambda = L_p^{real} / L_p^{rand}$, L_p^{rand} is the mean shortest path length of 100 matched random networks.
**Normalized C_p (γ)**	$\gamma = C_p^{real} / C_p^{rand}$, C_p^{rand} is the mean clustering coefficient of 100 matched random networks.
**Small-worldness σ**	$\sigma = \lambda/\gamma$, A real network would be considered small world if $\gamma > 1$ and $\lambda \approx 1$.

Local nodal properties	
**Nodal Betweenness Centrality $B_{nodal}(i)$**	$B_{nodal}(i)$ is defined as the fraction of all shortest paths in the network G that pass through a given node i, The mathematical definition is presented in (2).

The mathematical definition of $B_{nodal}(i)$ is shown in Equation (2):

$$B_{nodal}(i) = \sum_{s \neq i \neq t \in G} \frac{e_{st}(i)}{e_{st}}$$

$e_{st}(i)$ denotes the number of shortest paths in network G between node s and node t, which pass through node i, while e_{st} denotes the total number of shortest paths in network G between node s and node t. $B_{nodal}(i)$ captures the effect of a node on the information flow between all other nodes in a network and is usually used to identify brain network hubs [89]. Node *i* was considered a brain hub if $B_{nodal}(i)$ was at least 1 standard deviation (SD) greater than the average nodal BC of the network (i.e., $B_{nodal}(i)$ > mean + SD) [41].

Supplementary Table S3. Compare with previous similar study

**Title**	**Disrupted Gray Matter Networks Associated with Cognitive Dysfunction in Cerebral Small Vessel Disease**	**Disrupted topological organization of resting-state functional brain networks in cerebral small vessel disease**
**Topic**	Topological changes of brain gray matter (GM) structural network between CSVD-c, CSVD-n and HC	Topological changes of brain functional connectivity (fc) network between CSVD-c, CSVD-n and HC
**Method**	Brain structure networks were constructed by using GM volumetric parameters	Resting-state functional MRI were used to construct individual whole-brain and ROI-level FC networks
**Results**	Both CSVD-c and CSVD-n patients showed increased Eglob/Eloc and decreased Lp in GM structural networks	Only CSVD-c patients showed decreased Eglob/Eloc/Cp and increased Lp in functional networks
	The GM structural networks of CSVD-c patients exhibited significantly altered nodal betweenness centrality (BC) in the DMN and sensorimotor-associated functional regions	The functional networks of CSVD-c patients exhibited significantly altered nodal BC in the DMN and attention, and visual-related functional areas
	Eglob, Eloc and Lp of GM structural networks were significantly correlated with the SDMT score in the CSVD patients	Altered nodal BC of the right anterior/posterior cingulate gyrus and left cuneus in functional networks were significantly correlated with the clinical cognitive parameters of CSVD patients

In **Supplementary Table S3**, a comparison is made between this study and previous ones. The previous research on functional networks of CSVD patients discovered a notable reduction in both global and local efficiency, as well as a significant increase in the shortest path length. Both studies revealed variations in nodal BC in different brain functional modules in CSVD-c patients. These changes in the brain's topological characteristics are linked to the patients' cognitive and executive functions.

Supplementary Table S4. The list of abbreviations

The list of abbreviations	
Abbreviation	Full name
GM	gray matter
CSVD	cerebral small vessel disease
CMBs	cerebral microbleeds
CSVD-c	CSVD patients with CMBs
CSVD-n	CSVD patients without CMBs
E_{glob}	global efficiency
E_{loc}	local efficiency
L_p	shortest path length
DMN	default mode network
MoCA	Montreal Cognitive Assessment
AVLT	Rey Auditory Verbal Learning Test
TMT	Trail Making Test
SCWT	Stroop Color and Word Test
SDMT	Symbolic Digital Modalities Testing
TMT (B-A)	the difference between TMT-B and TMT-A
WMHs	white matter hyperintensities
CI	cognitive impairment
AD	Alzheimer's disease
Aβ	amyloid-beta
WM	white matter
GMV	gray matter volume
VBM	voxel-based morphometry
MCI	mild cognitive impairment
SWI	Susceptibility-weighted imaging
CSF	cerebrospinal fluid
TIV	total intracranial volume
BC	betweenness centrality
AUC	area under the curve
ANOVA	one-way analysis of variance
LSD	least significant difference
PVS	enlarged perivascular spaces
OHE	overt hepatic encephalopathy
lvPPA	logopenic variant of primary progressive aphasia
FC	functional connectivity
